# Comparison of Flavonoid O-Glycoside, C-Glycoside and Their Aglycones on Antioxidant Capacity and Metabolism during In Vitro Digestion and In Vivo

**DOI:** 10.3390/foods11060882

**Published:** 2022-03-20

**Authors:** Liangqin Xie, Zeyuan Deng, Jie Zhang, Huanhuan Dong, Wei Wang, Banghuai Xing, Xiaoru Liu

**Affiliations:** 1 State Key Laboratory of Food Science and Technology, Nanchang University, Nanchang 330047, China; liangqinxie2021@outlook.com (L.X.); dengzy@ncu.edu.cn (Z.D.); 2 Institute for Advanced Study, Nanchang University, Nanchang 330031, China; 3 Laboratory Animal Science and Technology Center, Jiangxi University of Chinese Medicine, Nanchang 330004, China; 20091021@jxutcm.edu.cn (J.Z.); banghuaixing@163.com (B.X.); 4 College of Pharmacy, Jiangxi University of Chinese Medicine, Nanchang 330004, China; donghh@jxutcm.edu.cn (H.D.); weiwang202202@163.com (W.W.)

**Keywords:** flavonoid, O-glycoside, C-glycoside, aglycones, in vitro digestion, antioxidant activity

## Abstract

Flavonoids are well known for their extensive health benefits. However, few studies compared the differences between flavonoid O-glycoside and C-glycoside. In this work, flavonoid O-glycoside (isoquercitrin), C-glycoside (orientin), and their aglycones (quercetin and luteolin) were chosen to compare their differences on antioxidant activities and metabolism during in vitro digestion and in vivo. In vitro digestion, the initial antioxidant activity of the two aglycones was very high; however, they both decreased more sharply than their glycosides in the intestinal phase. The glycosidic bond of flavonoid O-glycoside was broken in the gastric and intestinal stage, while the C-glycoside remained unchanged. In vivo, flavonoid O-glycoside in plasma was more elevated than C-glycoside on the antioxidant activity; however, flavonoid C-glycoside in urine was higher than O-glycoside. These results indicate that differences of flavonoid glycosides and their aglycones on antioxidant activity are closely related to their structural characteristics and metabolism in different samples. Aglycones possessed higher activity but unstable structures. On the contrary, the sugar substituents reduced the activity of flavonoids while improving their stability and helping to maintain antioxidant activities after digestion. Especially the C-glycoside was more stable because the stability of the C–C bond is higher than that of the C–O bond, which contributes to the difference between flavonoid O-glycoside and C-glycoside on the absorption and metabolism in vivo. This study provided a new perspective for comparing flavonoid O-glycoside, flavonoid C-glycoside, and their aglycones on their structure–activity relationship and metabolism.

## 1. Introduction

Reactive oxygen species (ROS) are harmful byproducts produced by all aerobic organisms in the process of conventional oxygen metabolism, including the superoxide radical anion (·O^2−^), the hydroxyl radical (HO·), peroxyl radicals (ROO·), and others [1]. Excessive ROS production leads to increased oxidative stress level in vivo, which may induce aging and various chronic diseases, such as cardiovascular disease, diabetes, and cancer [2,3]. Antioxidant scavenging free radicals is an essential defense against free radical damage. Therefore, the search for effective antioxidants is crucial for delaying aging and the prevention of various chronic diseases. As the most important subclass of polyphenols, flavonoids are often mentioned and reported in many studies. Polyphenols are a large group of bioactive substances widely found in plants, including flavonoids, phenolic acids, lignans, and stilbenes [4,5]. Flavonoids are commonly found in fruits and vegetables and are considered excellent antioxidants [6]. Flavonoids are important secondary metabolites that exhibit a broad spectrum of biological activities, including antioxidant, anti-inflammatory, antiallergic, antimutation, antibacterial, anticancer, and hepatoprotective activities. They have also been widely acknowledged for their health-promoting roles in preventing chronic diseases, such as cardiovascular diseases and diabetes. Therefore, some flavonoids have been applied to develop nutraceuticals and medicines [7,8].

Flavonoids are synthesized from hydroxyl cinnamic acid (B ring) and malonyl residue (A ring) through a series of condensation reactions with a skeleton of C6-C3-C6, including flavones, flavonols, flavanones, isoflavones, flavan-3-ols, chalcones, and anthocyanidins, etc. In plants, most flavonoids exist in different modification forms, such as hydroxylation, methylation, acylation, and glycosylation, among which glycosylation is the most common modification form of flavonoids [9,10,11]. Almost all natural flavonoids exist in plants in O-glycosides or C-glycosides. O-glycosides are formed by attaching sugar to hydroxyl oxygen. While C-glycosides are sugar moieties combined directly to flavonoid backbone as C–C covalent bonds. Should they share the same in physical and chemical properties and activities? Compared with O-glycosides, fewer studies were focused on flavonoid C-glycosides in the diet, for they have seldom been separated and purified [12,13]. However, their importance should not be underestimated. Flavonoid C-glycosides have a wide range of benefits to human health, such as antidiabetic, anti-inflammatory, antiAlzheimer’s disease, antioxidant, antiviral, and anxiolytic effects [13,14].

It is reported that flavonoid O-glycosides, flavonoid C-glycosides, and their aglycones show significant differences in physical and chemical properties and activities. For example, compared with their aglycones, glycosides can improve the stability of compounds, increase water solubility, reduce toxic and side effects, and improve the specific targeting property of drugs. Moreover, in general, glycosylation can protect plant compounds from self-oxidation, although it may reduce the activity of the compounds [15]. Some researchers reported O-glycosides can hydrolyze into corresponding aglycones and show similar biological activities to aglycones. C-glycosides show more diversified biological activities than O-glycosides, because C–C covalent bonds are steadier and resistant to acid, alkali, and enzymatic hydrolysis [7,12,16]. However, just how exactly they differ in structure and activity relationships requires further research.

In vitro digestion is vital for understanding the stability and bioaccessibility and assessing the bioavailability and bioactivity of compounds [17]. Bioaccessibility refers to converting a compound into a potentially bioavailable form and changes in the distribution of bioactive compounds. Therefore, evaluating bioavailability is of crucial importance before assessing any potential health benefits of flavonoids. Among them, the structure of flavonoids is a vital factor affecting biological accessibility, such as glycosylation, including the type of glycosidic bond (O-glycoside or C-glycoside), the type and number of the sugar moiety, and the site of glycosylation [18].

Flavonoids possess high antioxidant activity in vitro, which may not be the case in vivo. The absorption, metabolism, and distribution of compounds should be considered [11]. Glycosides have been reported to possess better bioavailability than aglycones in vivo [19]. Flavonoid C-glycosides have different pharmacokinetics and biological activities from O-glycosides due to their extremely high structural stability [13]. Orientin and vitexin share similar structures, and it was reported that the antioxidant activity of orientin in vivo was significantly higher than that of vitexin because of a phenolic hydroxyl [20]. A similar structure–activity relationship was reported by Wen that an apigenin diglycoside possessed better cellular antioxidant activity than apigenin with the difference of a bioside [7]. These results prove that the structure plays a crucial role in the compounds’ activity. Prototype drugs are rapidly metabolized after oral administration, and the metabolites may exert higher biological activity. Therefore, it is also indispensable to study the compound’s metabolism in vivo. Furthermore, information on flavonoid C-glycoside metabolism is limited [21]. Since the mechanism and effects of glycosylation have not been fully elucidated, the research of flavonoid O-glycoside, C-glycoside and their aglycones on structural stability, activity, and metabolism in vitro and in vivo is still of great value [12].

**Figure 1:**
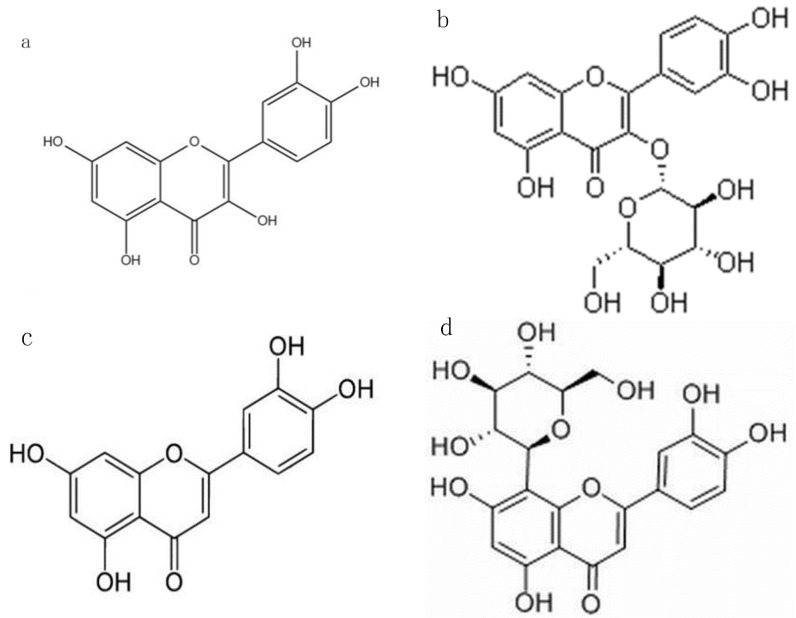
Structure of several flavonoids: (**a**) quercetin, (**b**) isoquercitrin, (**c**) luteolin, and (**d**) orientin.

The antioxidant effect of flavonoid compounds is the basis for their potential ability to prevent chronic diseases. The structural diversities of flavonoid compounds largely contribute to their different antioxidant activities [22]. Therefore, it is imperative to explore the relationship between flavonoid compounds, even their glycosides, on structures and antioxidant activity. Flavonoid O-glycosides, C-glycosides, and their aglycones present structural uniqueness and differences in physical and chemical properties, so the comparative study of their antioxidant activities is of unique value. Therefore, we selected the typical glycosylated flavonoids, O-glycoside (isoquercitrin), C-glycoside (orientin), and their aglycones (quercetin, luteolin), as shown in Figure 1, to explore their structure and activity relationship. Although they shared similar skeleton structures, does their content and antioxidant activity show some differences during in vitro digestion? Comprehensively, are there any differences among flavonoid O-glycoside, C-glycoside, and their aglycones on their metabolism and activity in vivo? In short, this work is constructive for understanding the differences among flavonoid O-glycoside, C-glycoside, and their aglycones in structural stability, bioavailability qualitatively, and antioxidant activity during in vitro digestion and in vivo quantitatively. These differences may be the key to exploring their physiological activities in the body. This study provided a new perspective for comparing flavonoid O-glycoside and flavonoid C-glycoside on their structures and antioxidant activity.

## 2. Materials and Methods

### 2.1. Selection of Flavonoid O-Glycoside, C-Glycoside, and Their Aglycones

Isoquercitrin is quercetin-3-O-glucoside, and orientin is luteolin-8-C-glucoside. They share a similar skeleton, while the remarkable difference lies in the glycosidic bond; isoquercitrin possesses a C–O bond, and orientin possesses a C–C bond. They were chosen to compare the differences between O-glycoside and C-glycoside. Glycosides are formed by aglycones and sugar moiety. Due to the modification of sugar moiety, isoquercitrin and orientin show different physical and chemical properties from their aglycones quercetin and luteolin. So, they were chosen to compare the differences between glycosides and aglycones.

### 2.2. Chemicals and Reagents

The standard substances isoquercitrin, orientin, and luteolin (all purity ≥ 98%) were purchased from Beijing Solarbio Technology Co., Ltd. (Beijing, China). Isoquercitrin (purity ≥ 98%) was provided by Wuhan Chemfaces Bio-Technology Co., Ltd. (Wuhan, China), and orientin (purity ≥ 98%) was purchased from Shanghai yuanye Bio-Technology Co., Ltd. (Shanghai, China) in animal experiments. Quercetin (purity ≥ 97%), 2,2-diphenyl-1-pi-Crylhydrazyl (DPPH), 2,2′-azinobis (3-ethylbenzothiazoline-6-sulfonic acid) diammonium salt (ABTS), 2,4,6-tris (2-pyridyl)-S-triazine (TPTZ), 2,2′-azobis (2-methylpropionamidine) dihydro-chloride (AAPH), fluorescein sodium, 6-hidroxy-2,5,7,8-tetramethylchroman-2-carboxylic acid (Trolox), α-amylase (500 U/g), pepsin (≥2500 U/mg), trypsin (≥3000 U/mg), and formic acid (HPLC gradient grade) were purchased from the Aladdin Reagent Co., Ltd. (Shanghai, China). In addition, bile salt was obtained from Beijing Solarbio Technology Co., Ltd. (Beijing, China). Ultrapure water was purchased from Watsons (Hongkong, China). Acetonitrile (HPLC gradient grade) was provided by Thermo Fisher Scientific (Waltham, MA, USA). T-AOC kit was purchased from Nanjing Jiancheng Bioengineering Institute (Nanjing, China) and Beyotime Biotechnology (Shanghai, China).

### 2.3. In Vitro Digestion

The samples’ digestion was digested according to the detailed recommendation of the standardized in vitro digestion method published by Minekus et al. with some modifications [18,23,24]. The scheme consisted of three digestions, which included oral, gastric, and intestinal digestion. The samples were mixed with simulated salivary fluid (SSF) in the oral digestion stage. Salivary α-amylase was added to achieve 75 UmL^−1^ in the ultimate mixture, by adding CaCl_2_ to reach 0.75 mM in the ultimate mixture, and the necessary amount of water was added to dilute the stock solution of SSF. The mixture was shaken on a water bath shaker (120 rpm, 37 °C) for 2 min. In the gastric digestion stage, the oral stage samples were mixed with simulated gastric fluid (SGF); porcine pepsin was added to achieve 2000 UmL^−1^ in the ultimate digestion mixture; CaCl_2_ was added to reach 0.075 mM in the ultimate digestion mixture, followed by 1 M HCl to reduce the pH to 3.0. The mixture was shaken on a water bath shaker (120 rpm, 37 °C) for 2 h. In small intestinal digestion stage, the gastric digestion stage samples continued to mix with simulated intestinal fluid (SIF); pancreatin was added to achieve 100 UmL^−1^ in the final digestion mixture; bile salts were added to give a final concentration of 10 mM in the final mix; CaCl_2_ was added to reach 0.3 mM in the final digestion mixture, and 1 N NaOH was added to reduce the pH to 7.0. The mixture was shaken on a water bath shaker (120 rpm, 37 °C) for 2 h.

### 2.4. Antioxidant Activity Assays during In Vitro Digestion

#### 2.4.1. DPPH Radical Scavenging Activity (DPPH Assay)

The DPPH free radical scavenging activity of samples was determined in 96-well microliter plates according to Ma et al. with some modifications [25,26]. The sample (20 μL) and DPPH reagent (0.26 mM, 100 μL) were mixed and kept in the dark for 30 min at room temperature, and the absorbance at 517 nm was recorded. The absorbance of the DPPH solution plus methanol was recorded as A_0_; the DPPH solution plus the sample was recorded as A_1_, and methanol (solvent) plus the sample was recorded as A_2_. The DPPH radical scavenging rate was calculated according to the equation: DPPH radical scavenging rate (%) = [A_0_ − (A_1_ − A_2_)]/A_0_ × 100%. The results are expressed as mM trolox equivalents (TE) per gram of sample (mM TE/g).

#### 2.4.2. ABTS Radical Scavenging Activity (ABTS Assay)

The samples ABTS free radical scavenging activity was determined according to a published method with some modifications [26]. The 5 mL 7.4 mM ABTS solution and 88 μL 140 mM K_2_S_2_O_8_ solution were mixed in the dark for 12–16 h at room temperature. The hybrid solution was diluted with 80% ethanol as the ABTS reagent (the absorbance at 734 nm adjusted to 0.700 ± 0.02). Briefly, the sample (20 μL) was mixed with ABTS reagent (200 μL) and kept in the dark for 6 min at room temperature, and the absorbance was measured at 734 nm. The absorbance of the ABTS solution plus 80% ethanol was recorded as A_0_; the ABTS solution plus the sample was recorded as A_1_, and 80% ethanol (solvent) plus the sample was recorded as A_2_. The ABTS radical scavenging rate was calculated according to the equation: ABTS radical scavenging rate (%) = [A_0_ − (A_1_ − A_2_)]/A_0_ × 100%. The results are expressed as mM trolox equivalents (TE) per gram of sample (mM TE/g).

#### 2.4.3. Ferric Ion Reducing Antioxidant Power (FRAP Assay)

The ferric reducing activities of the samples were determined according to the method described by Ma [25], with some modifications. The sample (10 μL) was mixed with fresh FRAP reagent (300 μL. The 300 mM acetate buffer pH 3.6, 10 mM TPTZ in 40 mM HCl, and 20 mM FeCl_3_·6H_2_O were mixed according to 10:1:1 as FRAP reagent) and reacted in the dark for 4 min. The absorbance of the mixture at 593 nm was then read. The results are expressed as mM trolox equivalents (TE) per gram of sample (mM TE/g).

#### 2.4.4. Oxygen Radical Absorbance Capacity (ORAC Assay)

The ORAC assay measures the antioxidant scavenging function against peroxyl radical-induced by AAPH at 37 °C [7,27]. In the ORAC assay, fluorescein sodium salt is used as a fluorescent probe with an excitation wavelength of 485 nm and an emission wavelength of 528 nm. The loss of fluorescein fluorescence indicates the extent of its reaction with the peroxyl radicals. All solutions were prepared in a phosphate buffer (75 mM, pH 7.4). The sample (25 μL) and fluorescein solution (8.68 × 10^−^^5^ mM, 150 μL) were mixed and incubated at 37 °C for 30 min. After the incubation, 25 μL AAPH (153 mM) was added quickly to start the reaction. Microliter plate fluorescence was recorded every minute for 120 min. Blank was phosphate buffer (75 mM, pH 7.4), and various concentrations of trolox (6.25–200 μM) were used as standards. The final ORAC values were calculated by standards or samples and the net area under the curve (AUC), subtracting the AUC of the blank. The results are expressed as mM trolox equivalents (TE) per gram of sample (mM TE/g).

### 2.5. HPLC–DAD Analysis

Agilent 1260 series HPLC system (Agilent Technologies, Santa Clara, CA, USA) equipped with an Agilent Zorbax Eclipse XDB-C18 column (250 mm × 4.6 mm, 5 μm) was used for the analysis of flavonoids. The samples were eluted with 0.1% formic acid water (*v*/*v*, solvent A) and acetonitrile (solvent B) as follows: 0–10 min, B linearly increased from 10% to 20%; 10–18 min, B maintained at 20%; 18–25 min, B linearly increased from 20% to 30%; 25–40 min, B linearly increased from 30% to 60%. The column temperature was held at 30 °C; the injection volume was 10 μL; the flow rate was 0.6 mL/min, and the UV detection was at 254 nm.

### 2.6. Animal Experiment

Male Sprague–Dawley rats (No. SCXK (XIANG) 2019-0004), weighing approximately 200–220 g, were purchased from Hunan SJA Laboratory Animal Co., Ltd. (Hunan, China). The rats were kept in standard conditions with the temperature 22–26 °C and relative humidity was 40%–60%. During the adaptive feeding period, the rats had free access to food and water. Animal experiments were conducted with the standard ethical guidelines of the Laboratory Animal Management Committee of Jiangxi Province (SYXK (GAN) 2017-0004).

Following three days of adaptive feeding, the rats were administered after fasting for 12 h. A total of twenty-eight rats were randomly divided into four groups, respectively, blank, quercetin, isoquercitrin, and orientin, with seven rats in each group. Rats in each group were orally administered the corresponding drugs at a dose of 250 mg/kg. In each group (*n* = 7), blood was collected from four rats, and urine was collected from the other three rats. Blood was collected in heparinized tubes after oral administration of samples at 1, 3, 6 h via the orbital vein and then centrifuged at 4000 rpm (4434× *g*) for 10 min at 4 °C to obtain the plasma. The other three rats were maintained in metabolic cages separately, and the urine was collected within 0–6 h, 6–12 h, and 12–24 h after oral administration of samples. All samples were stored at −80 °C.

### 2.7. Total Antioxidant Capacity Assay (T-AOC) In Vivo

The total antioxidant capacity of plasma and urine at different periods was measured according to the manufacturer’s instructions by the test kits (Nanjing Jiancheng Bioengineering Institute, Nanjing, China; Beyotime Biotechnology, Shanghai, China).

### 2.8. Q Exactive Analysis

Thermo Q Exactive UHMR Hybrid Quadrupole-Orbitrap Mass Spectrometer (Thermo Fisher Scientific, Waltham, MA, USA) equipped with a Thermo Accucore aQ column (150 mm × 2.1 mm, 2.6 μm) was used to analyze metabolites in plasma and urine. The samples were eluted with 0.1% formic acid water (*v*/*v*, solvent A) and acetonitrile (solvent B) as follows: 10–30% B from 0 to 5 min, 30–50% B from 5 to 10 min, 50–70% B from 10 to 20 min, 70–100% B from 20 to 30 min, 100% B from 30 to 35 min and 100–10% B from 35 to 37 min. The column temperature was held at 30 °C; the injection volume was 5 μL, and the flow rate was 0.3 mL/min. Mass spectra were obtained under the following conditions: range, *m*/*z* 100–1200; positive and negative ion modes; the collision energy was 20 and 40 eV. Compound Discoverer 3.1 software was used for data analysis.

A total of 100 μL plasma plus 10 μL formic acid vortexed for 1 min, then 300 μL acetonitrile was added and vortexed for 3 min. A total of 1 mL urine plus 50 μL formic acid was vortexed for 1 min; then, 8 mL ethyl acetate was added and vortexed for 3 min. The supernatant was obtained by centrifugation at 5000× *g* for 10 min at 4 °C and concentrated to dryness by nitrogen blowing. The dry residues were dissolved in 200 μL methanol.

### 2.9. Statistical Analysis

The results are displayed as means ± standard deviations (M ± SD). Statistical analysis was performed by SPSS, version 17.0. The analysis of variance was performed using one-way analysis of variance (ANOVA), and differences between the means of samples were analyzed by Duncan’s test and considered significance at *p* ≤ 0.05.

## 3. Results and Discussion

### 3.1. Antioxidant Activity of Flavonoids In Vitro Digestion

#### 3.1.1. DPPH Assay of Flavonoids

**Figure 2:**
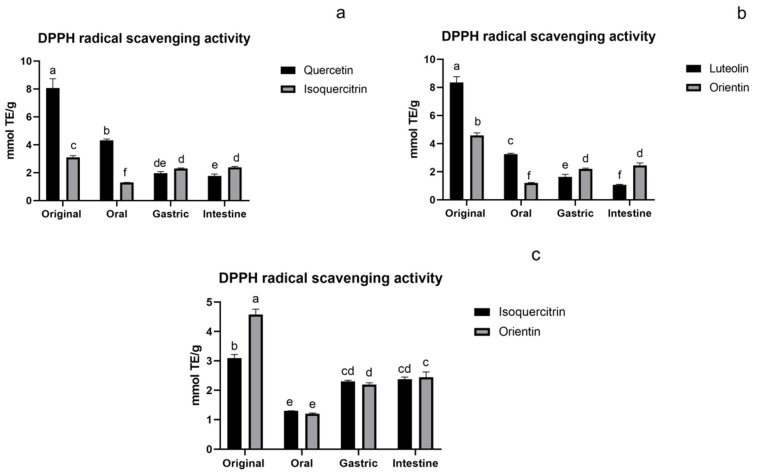
DPPH assay of flavonoids: (**a**) flavonoid O-glycoside and its aglycone, (**b**) flavonoid C-glycoside and its aglycone, and (**c**) flavonoid O-glycoside and flavonoid C-glycoside. The results are reported as means ± SD. Different letters in the figure indicate significant differences (*p* < 0.05).

The DPPH assay result of flavonoids during in vitro digestion are shown in Figure 2. In a and b, the original antioxidant activity of aglycones (both quercetin and luteolin) were very high. However, during the digestion process, they were significantly reduced and gradually lowered than their glycosylated compounds (both isoquercitrin and orientin). This was consistent with the results reported by Lee [28]. Because the instability of the aglycones leads to a rapid decline in contents during the digestion process, their activity was rapidly reduced. For isoquercitrin (O-glycoside) and orientin (C-glycoside), they maintained a relatively high activity compared to their original activity and were significantly higher than their aglycones in the intestinal stage. Glycosylation will reduce the activity while improving the stability of the compound. Therefore, glycosides can easily maintain relatively stable activity in vitro digestion. It showed that the influence of glycosylation on the stability of flavonoids is significant. It can be seen from Figure 2c that the activity of O-glycoside and C-glycoside showed no significant difference during digestion. The activity of flavonoid glycosides decreased in the oral phase and increased in the gastric and intestinal phases. Similar variations were found in bamboo leaf soup, which is full of flavonoids [25,29]. This may be due to the fact that the content of flavonoids decreased a lot in the oral digestion phase, thus, leading to the reduction of antioxidant activity. In the gastric and intestine phases, the content of flavonoids tended to be stable. Meanwhile, the acidic environment in gastric digestion was conducive to the stability of flavonoids [30], consequently, the content and activity of flavonoids were improved.

#### 3.1.2. ABTS Assay of Flavonoids

**Figure 3:**
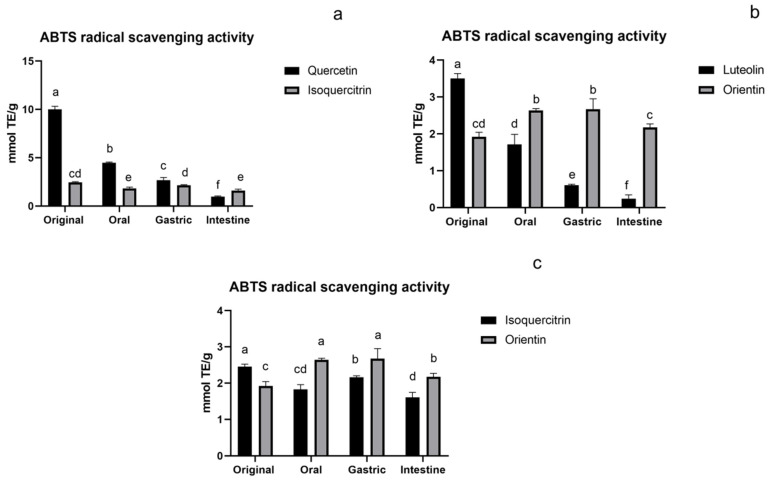
ABTS assay of flavonoids: (**a**) flavonoid O-glycoside and its aglycone, (**b**) flavonoid C-glycoside and its aglycone, and (**c**) flavonoid O-glycoside and flavonoid C-glycoside. The results are reported as means ± SD. Different letters in the figure indicate significant differences (*p* < 0.05).

As for the ABTS assay (Figure 3), in original, the aglycones’ (both quercetin and luteolin) activity were significantly higher than their glycoside compounds (isoquercitrin and orientin), but their activity decreased sharply during the digestion process and was even considerably lower than their glycosides in the intestine as shown in Figure 3a,b, which was similar to the DPPH assay. However, compared with their original activity, flavonoid O-glycoside and C-glycoside maintained vigorous antioxidant activity in the oral, gastric, and intestinal stages, especially flavonoid C-glycoside. The antioxidant activity of C-glycoside (orientin) was significantly increased during the digestion process than in the initial stage. Similar results were reported by Bouayed and Lucas that the ABTS activity of Jonagold apple and the chyme soluble fractions of spaghetti increased during in vitro digestion [31,32]. This may lie in the interaction between the digestive system and compound, which also helps to scavenge ABTS radicals. The activity of flavonoid C-glycoside was significantly higher than that of flavonoid O-glycoside during the digestion process, as shown in Figure 3c. This may be a reflection of the more stable structure of flavonoid C-glycoside, which made it easier to maintain higher activity.

#### 3.1.3. FRAP Assay of Flavonoids

**Figure 4:**
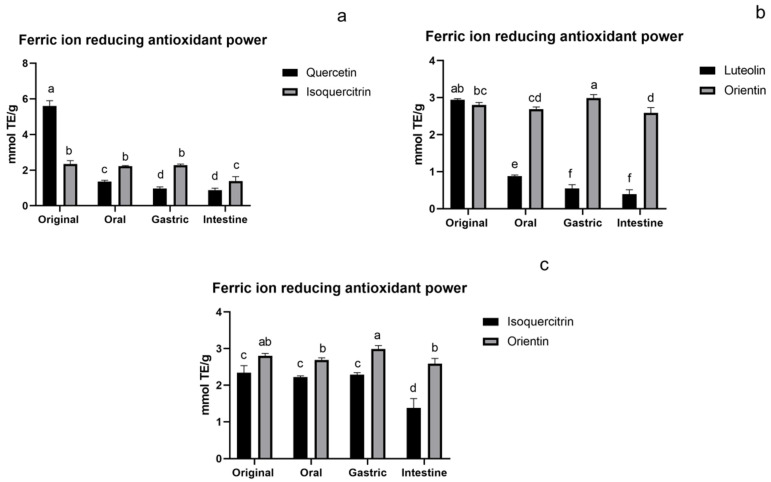
FRAP assay of flavonoids: (**a**) flavonoid O-glycoside and its aglycone, (**b**) flavonoid C-glycoside and its aglycone, and (**c**) flavonoid O-glycoside and flavonoid C-glycoside. The results are reported as means ± SD. Different letters in the figure indicate significant differences (*p* < 0.05).

For the FRAP assay, it can be seen from Figure 4 that it tended to be consistent with the changes of the ABTS assay. The ABTS activity had also been reported by Dudonné [33] to strongly correlate with FRAP activity. The antioxidant activity of aglycones decreased significantly, while glycosides maintained an intense activity compared to their original activity, and their activity exceeded that of aglycones from oral to the intestine. The general activity trend of flavonoid O-glycoside and C-glycoside was similar. Both of them maintained relatively high activity in the oral and gastric stages, and slightly decreased in the intestinal stage. Decreased activity at the intestinal phase was also observed in flavonoid-rich bamboo leaf soup and persimmon [25,34]. During the digestion process, the activity of flavonoid C-glycoside was significantly higher than that of O-glycoside, which was consistent with the ABTS assay.

#### 3.1.4. ORAC Assay of Flavonoids

**Figure 5:**
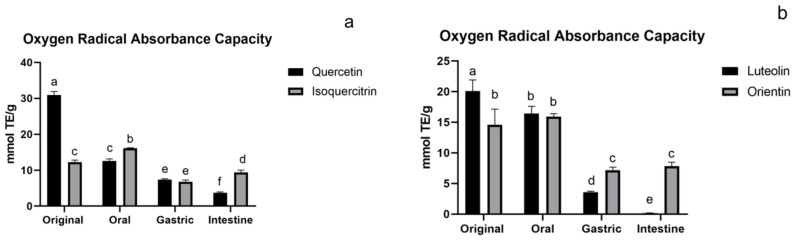

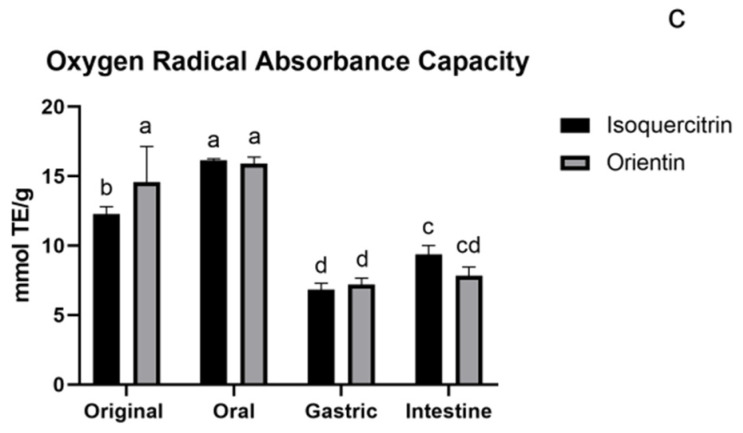
ORAC assay of flavonoids: (**a**) flavonoid O-glycoside and its aglycone, (**b**) flavonoid C-glycoside and its aglycone, and (**c**) flavonoid O-glycoside and flavonoid C-glycoside. The results are reported as means ± SD. Different letters in the figure indicate significant differences (*p* < 0.05).

The ORAC assay introduces kinetic parameters to evaluate the influence of the entire reaction process on the results, which is a more accurate method for assessing antioxidant activity. From Figure 5a,b, the aglycones activity decreased in turn and were gradually lowered than their glycoside compounds. The ORAC assay (Figure 5c) showed a different change from the first three assays. The activity was the highest in the oral stage and decreased in the gastric and intestinal stages. The changing trend of flavonoid O-glycoside and flavonoid C-glycoside tended to be the same. The grape pomace and skin of white wine byproducts also had a similar trend [35]. There was no significant difference in flavonoid O-glycoside and flavonoid C-glycoside during digestion. The ORAC had a low correlation with other methods, which is consistent with the report of Huang [36]. This is mainly because the ORAC has a different mechanism from the other three assays. The DPPH radical scavenging activity of apigenin was reported to be much less than that of quercetin, but their ORAC values were similar [37].

From the above analysis, the activity changes of flavonoid O-glycoside, flavonoid C-glycoside, and their aglycones were varied from several different evaluation methods, but the general trend was a coincidence. The original activity of glycosylated compounds was significantly lower than their aglycones, while during the digestion process in vitro, the activity turned out to be substantially higher than their aglycones. The glycosylation of flavonoids will reduce the aglycones’ activity but improve their stability and solubility. Therefore, the glycosylated compounds were more stable and maintained higher contents in the process of digestion. As a result, their activity was maintained easily. However, the activity of aglycones was lost rapidly without the modification of sugar moiety. There was no significant difference in their activities in the DPPH assay and the ORAC assay for flavonoid O-glycoside and C-glycoside. In contrast, in the ABTS assay and FRAP assay, the activity of C-glycoside was significantly higher than that of O-glycoside. This may be because the stability of flavonoid C-glycoside is more robust than that of flavonoid O-glycoside, so they maintained activity better. The activity changes of flavonoid O-glycoside and flavonoid C-glycoside in the DPPH, ABTS, and FRAP assays were generally same and were highest in the gastric, but slightly lower or equal in oral and intestine, which agreed with the results reported by Huang [36]. It was said that apple polyphenols were stable in the gastric [38], and apple polyphenols were mainly released in the gastric [31]. This may because the acidic environment in the gastric was conducive to flavonoids. The DPPH, ABTS, and FRAP assays shared excellent correlation, while the ORAC assay exhibited a weak correlation with the other three assays, which was mainly because the four antioxidant evaluation methods have different reaction mechanisms. The DPPH, ABTS, and FRAP assays are generally based on the single electron transfer (SET) mechanism. In contrast, the hydrogen atom transfer (HAT) mechanism is the primary reaction mechanism of the ORAC assay, which may also involve sequential proton-loss electron transfer (SPLET), single-electron transfer followed by proton transfer (SET-PT), and sequential proton-loss hydrogen-atom transfer (SPLHAT) mechanism [39,40]. On the other hand, different solvents and pH conditions in the four antioxidant reactions also cause differences. Bouayed et al. hypothesized that FRAP was more suitable for evaluating antioxidant activity in gastric, while the other three assays were more appropriate to assess in intestine [31]. Therefore, a comprehensive evaluation of various antioxidant methods could objectively reflect the antioxidant activity of compounds. In conclusion, for flavonoid O-glycoside, C-glycoside, and their aglycones, their structural characteristics lead to changes in content during in vitro digestion (Table 1), and then lead to changes in activity, namely, the structure affects the activity of the compounds.

### 3.2. HPLC Analysis of Flavonoids during In Vitro Digestion

**Figure 6:**
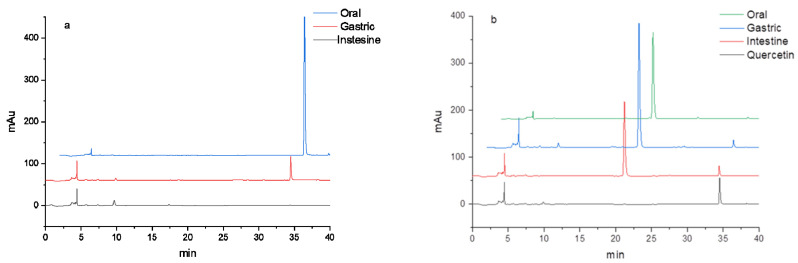

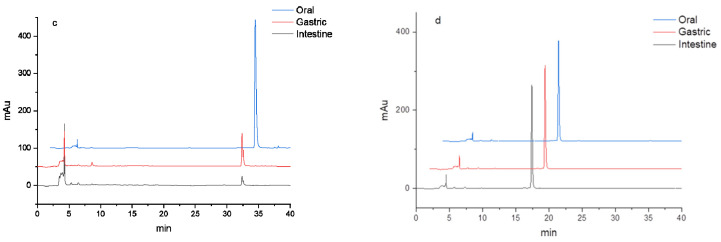
HPLC profiles of digested flavonoids: (**a**) quercetin, (**b**) isoquercitrin, (**c**) luteolin, and (**d**) orientin.

To detect the content changes of flavonoid O-glycoside, C-glycoside, and their aglycones during in vitro digestion, the digested compounds were analyzed by HPLC. The results are shown in Table 1 and Figure 6. It was shown that the contents of two aglycones, quercetin and luteolin, decreased rapidly after digestion in the oral, gastric, and intestine and were even reduced beyond the detection limit in the intestine. This was mainly because aglycone compounds were unstable and degraded rapidly due to digestive enzymes and digestive fluid, which were also consistent with their activity changes. Pellegrini reported that the content of quercetin decreased significantly during digestion [41]. At the same time, O-glycoside isoquercitrin and C-glycoside orientin maintained relatively high contents during digestion, confirming the stability of glycosylated compounds. Because the additional sugar moiety can protect the aglycones from degradation to some extent [11]. Xiang et al. also reported that the stability of myricitrin was greater than that of myricetin [42]. In the gastric and intestine, the quercetin could be detected in isoquercitrin by HPLC, which proved that the O-glycoside was broken down, and the isoquercitrin was deglycosylated into the aglycone quercetin. However, there was no luteolin detected in orientin. O-glycoside was easily damaged by hydrolysis. However, C-glycoside is a more stable structure, and the C–C covalent bond is hard to break. The content of C-glycoside in the intestinal stage was higher than that of O-glycoside, which also reflected that the stability of C-glycoside was higher than that of O-glycoside. In general, the content changes of O-glycoside, C-glycoside, and their aglycones during in vitro digestion correspond to their activity changes, which is related to their respective structural characteristics.

### 3.3. Analysis of Total Antioxidant Capacity and Metabolism of Flavonoids in Rat Plasma and Urine

**Figure 7:**
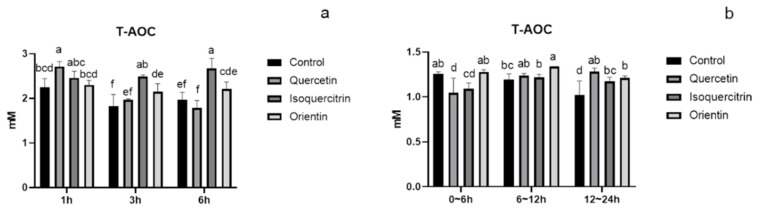
Changes of total antioxidant capacity of flavonoids in plasma and urine: (**a**) plasma and (**b**) urine. The results are reported as means ± SD. Different letters in the figure indicate significant differences (*p* < 0.05).

Flavonoid O-glycoside, C-glycoside, and their aglycones on the changes of total antioxidant capacity in plasma and urine are shown in Figure 7, and their metabolites are shown in Table 2, Table 3 and Table 4. In plasma (Figure 7a), flavonoid O-glycoside (isoquercitrin) was significantly higher than its aglycone (quercetin) and flavonoid C-glycoside (orientin) on the antioxidant activity at 3 h and 6 h. In urine (Figure 7b), flavonoid C-glycoside (orientin) was significantly higher than flavonoid O-glycoside (isoquercitrin) on the antioxidant activity at 0~6 h and 6~12 h, while, there was no significant difference between the activity of O-glycoside (isoquercitrin) and its aglycone (quercetin). As shown in Table 2 and Table 3, the metabolism of isoquercitrin and quercetin shared some similarities. They were metabolized through phase I reactions, including hydration, oxidation, dehydration, and desaturation and phase II reactions that included methylation, sulfation, and glucuronidation in vivo [43]. While flavonoid O-glycoside isoquercitrin was metabolized more sufficiently, more abundant metabolites could be detected (Table 3). These metabolites contributed to some essential physiological activities, including antioxidant activity [8,43,44,45]. In contrast, flavonoid C-glycoside was poorly absorbed, so there were few metabolites in the plasma and urine (Table 4). As a result, flavonoid O-glycoside in plasma was higher than flavonoid C-glycoside on the antioxidant activity. In urine, flavonoid O-glycoside (isoquercitrin) had been catabolized, while the prototype compound of flavonoid C-glycoside (orientin), could still be detected, which contributed to its antioxidant activity. Therefore, flavonoid C-glycoside in urine was higher on antioxidant activity than that of flavonoid O-glycoside. It had been reported that the recovery rate of flavonoid C-glycosides in 24 h was more than 20% [46] and that of vitexin glycosides in 24 h was more than 60% [47]. It was also suggested that flavonoid C-glycosides can maintain their prototype for a long time because of the stable chemical structure, which was conducive to their antioxidant activity and other benefits in vivo. It was reported that isoorientin (isomer of orientin) had a favorable half-life, and it was possible to maintain an effective concentration in vivo for a moderately long time to achieve the purpose of intravenous administration to treat diseases. Compared with the aglycone quercetin, O-glycoside isoquercitrin processes better bioavailability because sugar modification improves the solubility and stability of aglycone [44,48]. This contributes to its higher plasma concentrations and longer residence time on average and may help to exert physiological activity in vivo. Isoquercitrin can release quercetin through the cleavage of a glycosidic bond, which may exert the physiological activity of quercetin (Table 3). In conclusion, flavonoid O-glycoside, C-glycoside, and aglycones exhibited many differences in their structures and antioxidant activity relationships as well as metabolism. The differences in long-term antioxidant activities of flavonoid O-glycosides, C-glycosides, and their aglycones need further research. In addition to the typical glucose monoglycosides selected in this study, the type, quantity, and site of sugar moiety would also affect the physicochemical properties and activities of flavonoids. In short, the effect of glycosylation on flavonoids is multifaceted and complex.

## 4. Conclusions

Flavonoid O-glycoside, C-glycoside, and their aglycones were compared on antioxidant activity and metabolism during in vitro digestion and in vivo by selecting the typical and cognate structured isoquercitrin, orientin, and their aglycones, quercetin and luteolin. The results indicate that the flavonoid C-glycoside was higher than or equal to flavonoid O-glycoside on antioxidant activity during in vitro digestion. Compared with its aglycones, the antioxidant activity of glycosides is relatively stable and becoming higher than its aglycones in the process of digestion. Overall, the differences of structural stability of flavonoid O-glycoside, C-glycoside, and their aglycones lead to their content changes during in vitro digestion, resulting in differences in their activity. In vivo, flavonoid O-glycoside in plasma was higher than flavonoid C-glycoside on antioxidant activity, while in urine, flavonoid C-glycoside was higher than O-glycoside, which was due to their different absorption and metabolism in vivo. Although there have been many studies on these compounds, few studies compared them on O-glycoside and C-glycoside structure. Our study provides essential data to inspect the differences of flavonoid O-glycoside, C-glycoside, and their aglycones on antioxidant activity and metabolism during in vitro digestion and in vivo. At the same time, it is of great importance to understand flavonoid O-glycoside, C-glycoside, and their aglycones when it comes to the relationship between the structure and activity.

## Figures and Tables

**Table 1:** The contents of compounds during in vitro digestion.

Compounds	Stage (Content mg/g)			
	Original	Oral	Gastric	Intestine
Quercetin	1.00 ^a^	0.45 ± 0.12 ^b^	0.04 ± 0.01 ^c^	ND
Isoquercitrin	1.00 ^a^	0.65 ± 0.13 ^bc^	0.73 ± 0.08 ^b^	0.54 ± 0.08 ^c^
Luteolin	1.00 ^a^	0.84 ± 0.11 ^b^	0.13 ± 0.02 ^c^	ND
Orientin	1.00 ^a^	0.67 ± 0.01 ^b^	0.68 ± 0.10 ^b^	0.74 ± 0.03 ^b^

Note: Different letters in the same line are significantly different according to the Duncan test (*p* < 0.05); ND: not detected.

**Table 2:** Identification of metabolites of quercetin in plasma and urine.

NO	t_R_/Min	[M − H]^−^ /[M + H]^+^	PPM	Fragment Ions (*m*/*z*)	Mode	Formula	Transformations	Location	
								P	U
1	1.247	377.053	4.21	301.038	Neg	C_17_H_14_O_10_	Hydration, Oxidat- ion, Acetylation	−	+
2	5.419	477.067	−1.83	301.035 151.002	Neg	C_21_H_18_O_13_	Glucuronide Conjugation	−	+
3	5.552	447.091	−2.92	153.017	Pos	C_21_H_18_O_11_	Dehydration, Gluc- oside Conjugation	−	+
4	5.644	477.067	−1.45	301.035 151.002	Neg	C_21_H_18_O_13_	Glucuronide Conjugation	−	+
5	5.712	447.091	−2.79	153.017	Pos	C_21_H_18_O_11_	Dehydration, Gluc- oside Conjugation	−	+
6	9.047	297.047	−1.83	151.057	Neg	C1_6_H_10_O_6_	Dehydration, Methylation	−	+
7	11.850	395.243	−2.64	329.230	Neg	C_16_H_12_O_10_ S	Methylation, Sulfation	−	+
8	16.501	315.181	−1.65	300.030	Neg	C_16_H_12_O_7_	Methylation	−	+
9	22.344	311.020	3.10	151.110	Neg	C_16_H_8_O_7_	Didesaturation, Methylation	+	+
10	24.500	329.233	−2.12	301.200 151.040	Neg	C_16_H_10_O_8_	Desaturation, Oxid- ation, Methylation	+	+
11	24.850	463.289	−1.98	151.280	Neg	C_21_H_20_O_12_	Glucoside Conjugation	+	−

Note: P: plasma; U: urine; “+”: detected; “−”: undetected.

**Table 3:** Identification of metabolites of isoquercitrin in plasma and urine.

NO	t_R_/Min	[M − H]^−^ /[M + H]^+^	PPM	Fragment Ions (*m*/*z*)	Mode	Formula	Transformations	Location	
								P	U
1	0.923	191.027	−4.53	151.005	Neg	C_6_H_8_O_7_	Desaturation, Oxidation	+	−
2	1.107	335.042	2.94	301.081 151.039	Neg	C_15_H_12_O_9_	Hydration, Oxidation	−	+
3	1.118	491.082	−1.71	315.051 300.027	Neg	C_22_H_20_O_13_	Desaturation, Oxida- tion, Methylation	−	+
4	1.127	191.027	−4.53	151.075	Neg	C_6_H_8_O_7_	Desaturation, Oxidation	+	−
5	1.135	477.075	−1.09	301.035 151.002	Neg	C_21_H_18_O_13_	Glucuronide Conjugation	−	+
6	1.185	477.067	−1.64	301.035 151.002	Neg	C_21_H_18_O_13_	Desaturation, Oxidation	−	+
7	1.263	491.082	−2.06	315.051 300.027	Neg	C_22_H_20_O_13_	Desaturation, Methylation	−	+
8	1.269	491.090	−1.96	315.051 300.027	Neg	C_22_H_20_O_13_	Desaturation, Oxida- tion, Methylation	−	+
9	4.406	653.099	−0.57	301.035 151.002	Neg	C_27_H_26_O_19_	Desaturation, Gluc- uronide Conjugation	+	+
10	4.474	655.113	−1.26	303.049	Pos	C_27_H_26_O_19_	Desaturation, Gluc- uronide Conjugation	+	+
11	5.987	491.083	−0.71	315.051 300.027	Neg	C_22_H_20_O_13_	Dehydration, Methylation	−	+
12	6.283	491.090	−1.32	315.051 300.027	Neg	C_22_H_20_O_13_	Desaturation, Oxida- tion, Methylation	−	+
13	7.810	329.233	−0.52	301.200 151.040	Neg	C_16_H_10_O_8_	Desaturation, Oxid- ation, Methylation	−	+
14	8.908	317.078	−1.26	153.018	Pos	C_16_H_12_O_7_	Methylation	−	+
15	11.800	395.201	0.38	380.080	Neg	C_16_H_12_O_10_ S	Methylation, Sulfation	−	+
16	14.530	315.058	−1.16	300.027 151.002	Neg	C_16_H_12_O_7_	Methylation	−	+
17	15.593	557.022	−3.15	301.036	Neg	C_21_H_18_O_16_ S	Desaturation, Sulfation	+	+
18	15.604	557.023	−3.15	301.059	Neg	C_21_H_18_O_16_ S	Desaturation, Oxida- tion, Sulfation	+	+
19	22.620	301.165	−3.94	151.004	Neg	C_15_H_10_O_7_	Deglycosylation	+	+
20	32.814	315.058	−2.81	300.027 151.003	Neg	C_16_H_12_O_7_	Methylation	−	+

Note: P: plasma; U: urine; “+”: detected; “−”: undetected.

**Table 4:** Identification of metabolites of orientin in plasma and urine.

NO	t_R_/Min	[M − H]^−^ /[M + H]^+^	PPM	Fragment Ions (*m*/*z*)	Mode	Formula	Transformations	Location	
								P	U
1	6.700	447.093	−0.67	357.135	Neg	C_21_H_20_O_11_		−	+
2	12.510	447.093	−0.99	357.240	Neg	C_21_H_20_O_11_		−	+
3	19.310	445.272	−0.74	297.250	Neg	C_21_H_18_O_11_	Dehydration	−	+
4	27.445	465.102	−4.52	357.736	Neg	C_21_H_22_O_12_	Hydration	+	−
5	27.445	465.101	−4.52	357.736	Neg	C_21_H_22_O_12_	Reduction	+	−
6	27.529	465.101	−4.06	357.736	Neg	C_21_H_22_O_12_	Reduction	+	−
7	27.529	465.102	−4.06	357.736	Neg	C_21_H_22_O_12_	Hydration	+	−

Note: P: plasma; U: urine; “+”: detected; “−”: undetected.

## Data Availability

Not applicable.
